# 2D morphometric analysis of *Arabidopsis thaliana* nuclei reveals characteristic profiles of different cell types and accessions

**DOI:** 10.1007/s10577-021-09673-2

**Published:** 2021-10-19

**Authors:** Penka Pavlova, Martijn van Zanten, Basten L. Snoek, Hans de Jong, Paul Fransz

**Affiliations:** 1 Laboratory of Genetics, Wageningen University and Research, Droevendaalsesteeg 1, 6708 PB Wageningen, The Netherlands; 2 Molecular Plant Physiology, Institute of Environmental Biology, Utrecht University, Padualaan 8, 3584 CH Utrecht, The Netherlands; 3 Theoretical Biology and Bioinformatics, Institute of Biodynamics and Biocomplexity, Utrecht University, Padualaan 8, 3584 CH Utrecht, The Netherlands; 4 Swammerdam Institute for Life Sciences, University of Amsterdam, Science Park 904, 1098 XH Amsterdam, The Netherlands

**Keywords:** Nuclear phenotype, Quantitative analysis, Chromocenter, Heterochromatin, Arabidopsis

## Abstract

Functional changes of cells upon developmental switches and in response to environmental cues are often reflected in nuclear phenotypes, showing distinctive chromatin states corresponding to transcriptional changes. Such characteristic nuclear shapes have been microscopically monitored and can be quantified after differential staining of euchromatin and heterochromatin domains. Here, we examined several nuclear parameters (size, DNA content, DNA density, chromatin compaction, relative heterochromatin fraction (*RHF*), and number of chromocenters) in relation to spatial distribution of genes and transposon elements (TEs), using standard 2D fluorescence microscopy. We provide nuclear profiles for different cell types and different accessions of *Arabidopsis thaliana*. A variable, yet significant, fraction of TEs was found outside chromocenters in all cell types, except for guard cells. The latter cell type features nuclei with the highest level of chromatin compaction, while their chromocenters seem to contain gene-rich regions. The highest number of parameter correlations was found in the accession Cvi, whereas L*er* showed only few correlations. This may point at differences in phenotype robustness between accessions. The significantly high association of NOR chromocenters in accessions Ws and Cvi corresponds to their low *RHF* level.

**Supplementary Information:**

The online version contains supplementary material available at 10.1007/s10577-021-09673-2.

## Introduction

Interphase nuclei of *Arabidopsis thaliana* display considerable morphological variation between cell types, developmental stages and accessions, and alters in response to external factors such as temperature, light and pathogen invasion (reviewed in Fransz and de Jong 2011; Schubert and Shaw, 2011; van Zanten et al. 2012; Zhu et al. 2015; Poulet et al. 2015; Kaiserli et al. 2018; Perrella et al. 2020). This variation can be described in terms of typical dense chromatin bodies, spatial locations of repeat sequences, epigenetic marks, and distributions of chromatin-associated proteins, including histone variants and their specific modifications. The characteristic nuclear phenotypes reflect the microscopically distinguishable classes of euchromatin and heterochromatin, originally defined by Heitz (1928). Later, the concept of heterochromatin was further extended by Brown (1966), who introduced the term “constitutive” heterochromatin to describe a permanent state of compaction, and “facultative” heterochromatin, referring to a more temporary state of heterochromatin, such as the inactivated X chromosome (Barr body) in female mammals.

Nowadays, euchromatin and heterochromatin are merely holistically characterized as chromatin states with specific epigenetic modifications associated with either active genes, repressed genes, transposable elements, or other repeats. Based on multiple profiles, a classification of chromatin subtypes has been proposed for *Drosophila* (Filion et al. 2010), human (Ernst et al., 2011), and *Arabidopsis* (Roudier et al. 2011, Sequeira-Mendes et al., 2014). Although numbers of chromatin subtypes vary between these model species, they share three main groups: (1) Euchromatin, which represents gene regions with predominantly active epigenetic marks, such as H3K4me3, H3K36me3 and histone acetylation; (2) repressive chromatin (also referred to as facultative heterochromatin), which contains mainly inactive genes, generally associated with H3K27me3 and Polycomb proteins; and (3) constitutive or C-heterochromatin, which is rich in transposons and other repetitive elements, and contains high levels of methylated H3K9 and DNA methylation (plant and human). Integration of genome-wide datasets on chromatin states in Arabidopsis, rice, and maize resulted in an extended plant chromatin state database (Liu et al. 2018).

Cytogenetic analyses of Arabidopsis nuclei stained with DNA binding fluorescent dyes have revealed characteristic patterns of chromatin types at the light microscopy level. Compact heterochromatin domains, known as chromocenters or CCs (Fransz et al. 2002), are easily identified as discrete, intensely DAPI fluorescing domains at the periphery of the nucleus and are rich in H3K9me2 and methylated DNA (Soppe et al. 2002). Such typical chromocenter patterns have been observed in dicotyledon plant species with a genome size less than 1 Gb (Ceccarelli et al. 1998; Houben et al. (2003), analyzed 24 plant species with genome sizes ranging from 170 Mb (*Arabidopsis*) to 43 Gb (*Trillium*) to assess the relation between H3K9 methylation distribution and distinct heterochromatin morphology. In the species with up to around 500 Mb (all of them dicotyledon species), the methylated H3K9 was confined to microscopically detectable heterochromatin. Rice (490 Mbp) and other (mostly monocotyledon) plant species with larger genome size exhibited dispersed labelling of the H3K9me along the entire chromatin. Euchromatin shows a more uniform pattern enriched in H3K4me3 and acetylated histones throughout the nucleus, except for the chromocenters and the nucleolus (Fransz et al. 2002; Houben et al. 2003). Repressive facultative heterochromatin is visible throughout the euchromatin region as densely speckled areas enriched with H3K27me3 and LIKE HETEROCHROMATIN PROTEIN 1 (LHP1) (Lindroth et al. 2004; Mathieu et al. 2005; Naumann et al. 2005; Libault et al. 2005).

The distinct structures of the chromocenters provide easily quantifiable parameters for swift microscopic description of nuclear and chromatin phenotypes, which can be used to assess differences between cell types within a species, between accessions or between different species (Ceccarelli et al., 1998). To facilitate quantification of nuclear and chromatin morphometry, we introduced the term relative heterochromatin fraction (*RHF*), which represents the portion of total fluorescence intensity of all chromocenters relative to the fluorescence intensity of the entire nucleus (Soppe et al. 2002). Later on, Tessadori et al. (2007a) used the Heterochromatin Index (HX) to express the percentage of nuclei with a “normal” chromocenter appearance as fraction of the total population of assessed nuclei (that may include nuclei exhibiting decondensed, dispersed chromocenters). The *RHF* and HX parameters have been used since then to measure the dynamics of heterochromatin compaction during development and in response to biotic and abiotic stress conditions (Tessadori et al. 2007a, b; 2009; van Zanten et al. 2010, 2011; Pecinka et al. 2010; Bourbousse et al. 2015). A more sophisticated method was developed using 3D image processing technology for measuring the relative heterochromatin volume (RHV) and the position and distances between chromocenters (Andrey et al. 2010; Poulet et al. 2015, 2017; Ashenafi and Baroux, 2018; Desset et al. 2018; Arpòn et al. 2018). However, this accurate computational analysis of the 3D nuclear phenotype is in some cases less applicable for morphometric analysis, due to the time-consuming acquisition and analysis of the confocal images.

Extensive nuclear profiling of 2D cell spread images has been applied for the analysis of different chromocenter morphometric parameters, such as area, perimeter, density, roundness, and *heterogeneity*, to establish genetic variation between the *Arabidopsis thaliana* L*er* and Cvi accessions and a core population of 46 recombinant inbred lines (Snoek et al. 2017). The light and temperature receptor phytochrome B was subsequently confirmed after QTL analysis as a determinative factor for nuclear size and heterochromatin organization. In spite of these diverse studies on chromocenter and nuclear phenotypes, a detailed and systematic study of nuclear and chromatin organization in different cell types across different accessions was however still lacking.

In this study, we focus on parameters that are related to the distribution of genomic DNA and heterochromatic sequences and include *size*, *DNA content*, *DNA density*, *RHF*, and *number of chromocenters (CCs)*. We performed morphometric analysis of interphase nuclei in different cell types and organs of five *Arabidopsis thaliana* accessions. Based on the nuclear phenotypes, we created specific nuclear profiles for the different cell types and accessions. Such profiles shed light on the nuclear distribution of genomic elements, including the compaction of gene regions in chromocenters of guard cells and the decondensation of almost all transposons in parenchyma and pavement cells of Cvi, as well as in large nuclei of Col. This study shows that with basic tools and equipment, it is possible to quantify nuclear features for a better understanding of heterochromatin-related processes.

## Materials and methods

### Plant material

The *Arabidopsis thaliana* accessions Col (N1092), Landsberg *erecta* (L*er*) (NW20), Wassilewskija (Ws) (Ws-2), and Cvi were obtained from the Arabidopsis Biological Resource Stock Center (ABRC, Nottingham, UK). We also used a C24 accession-derived transgenic line, carrying H_2_B-YFP construct that was kindly provided by F. Berger. Seeds were stratified at 4 °C for 3 days before sawing and were grown in a climate chamber under white fluorescent light (180 µmol/m^2^/s) in long-day photoperiods of 16 h light/8 h dark, and constant temperature (23 °C/18 °C day/night) and humidity (70%). Whole plantlets were harvested at different developmental stages, starting from stage 1.04 to 9.70 according to Boyes et al. (2001) and were then fixed with ice-cold freshly prepared ethanol/acetic acid (3:1). The material was transferred to 70% ethanol after 24 h and stored at − 20 °C until further use.

### Slide preparation

Cell spread preparations were made according to the protocols of Soppe et al. (2002) and Pavlova et al. (2010). Rosette leaves and other plant organs fixed in Carnoy’s solution (ethanol/acetic acid 3:1) were washed 3 × 5 min with Milli-Q water and 1 × 5 min with 10 mM Na-citrate buffer (pH7.0) and then digested in a cocktail of pectolytic enzymes (Cytohelicase, Pall Life Sciences, Cellulose RS, Yakult Honsha Co., Ltd and Pectolyase Y23, Sigma) in a final concentration of 0.1% (for each enzyme) in Na-citrate buffer for 3 h at 37 °C. The digested material was transferred to water and chopped with a needle to a fine suspension. A droplet of 5 µL suspension was mixed with 20–40 µL 45% acetic acid and spread on a slide for 15 s on a hotplate at 43 °C. Extra washes in ice-cold acetic acid–ethanol (3:1) were carried out, followed by air-drying and storing at 4 °C. The nuclei were stained by 4’,6-diamidino-2-phenylindole (DAPI, 4 μg/mL), propidium iodide (PI, 4 μg/mL), or SYTOX® Green (35 nM, Invitrogen), and slides were mounted in Vectashield (Vector Laboratories, Burlingame, CA, USA) before observation. For the PI-stained slides, we incubated in 100 mg/mL RNAse A (Roche, the Netherlands) for 1 h at 37 °C prior to the DNA staining.

### FISH

Fluorescence in situ hybridization (FISH) experiments were carried out as described by Lysak et al. (2006) with slight modifications. Chromosome preparations were dried by overnight incubation at 37 °C. Slides were subsequently treated with RNAse (100 µg/mL in 2 × SSC) for 1 h at 37 °C and rinsed in 2 × SSC buffer for 3 × 5 min. Next, the material was fixed in 1% (w/v) (para)formaldehyde in PBS buffer (10 mM sodium phosphate buffer, pH 7.0, 143 mM NaCl) for 10 min and rinsed in 2 × SSC for 3 × 5 min, dehydrated through an ethanol series (70%, 90%, and 100%, each 2 min), and subsequently air-dried. To each slide 20 µL of hybridization mix, containing 100 ng probe in 50% formamide, 2 × SSC, 50 mM sodium phosphate (pH 7.0), and 10% sodium dextran sulfate, was added. The slide was subsequently denatured on a hot block set at 80 °C, for 2.5 min. The slides were incubated in a moist chamber at 37 °C for 18 h. Post-hybridization washes were performed in 50% formamide, 2 × SSC (pH 7.0) for 3 × 5 min at 42 °C, followed by 2 × SSC at room temperature for 3 × 5 min, dehydration through an ethanol series and staining with 2 µg/mL DAPI on slides. 45S rDNA was used as a probe (Gerlach and Bebrook, 1979) and directly labelled with DEAC-dUTPs using a standard Nick-translation kit (La Roche).

### Image acquisition and processing

#### Fluorescence microscopy

Slides were examined using a Zeiss Axioplan 2 Photomicroscope equipped with N.A. 1.4 Plan-Apochromatic objectives, epi-fluorescence illumination and appropriate small band filter sets for DAPI, propidium Iodide, Sytox Green, and DEAC fluorophores. We captured 12-bit raw images with a Photometrics Sensys 1305 × 1024 pixels CCD camera using the Genus Image Analysis Workstation software (Applied Imaging Corporation). Exposure of the images was set to control the full dynamic range of the image by moving the black and white point switches left and right of the image display curve for optimal contrast (black) and brightness (white). Images were saved as 8-bit RGB TIFF files for quantitative analysis with the freeware Mac OSX software Object Image (modified as https://www.quantitative-plant.org/software/objectj, the ImageJ plugin CHIAS (http://www2.kobe-u.ac.jp/~ohmido/index03.htm) and Image Pro Plus v.5 (http://www.mediacy.com/). FISH images were stored for each fluorescence signal separately and merged in Adobe Photoshop multilayer images using different blend modes (Kantama et al. 2017).

#### Confocal laser scanning microscopy

Twelve-bit images were recorded using an LSM 510 confocal laser scanning microscope (Carl Zeiss, Göttingen, Germany) equipped with a 63 × /1.4 NA Plan Apochromatic objective. We used an Argon ion laser at 364 and 488 nm and a He/Ne laser at 543 nm for detecting DAPI (385–470 nm band pass filter) and FITC (505–530 nm band pass filter), respectively. Images were scanned as 512 by 512 × 9 voxel images with a sampling rate of 140 × 140 × 700 nm (*x*, *y*, *z*).

#### Image processing and analysis

In order to semi-automatically analyze high numbers of raw (unprocessed) images, we applied our in-house developed macro in ImagePro-Plus (Media Cybernetics, Silver Spring, MD, USA) for the morphometric analysis to segment the nuclei and measure size (number of pixels) and fluorescence intensity of the interphase nucleus and individual chromocenters by a threshold algorithm (Pavlova et al. 2010). We monitored the biological parameters *size*, *DNA density*, *DNA content*, *variation in DNA density*, *relative heterochromatin fraction* (*RHF*), and *number of chromocenters* (see Table 1 for detailed description). Thirty to one hundred nuclei per sample were examined in every experiment. Data were exported to a Microsoft Excel spreadsheet and further analyzed using the open-source statistical program JASP (JASP Team, 2020, Version 0.13.01, Computer software, https://jasp-stats.org). Comparisons were evaluated by a one-way *ANOVA* test or a *t*-test.

**Table 1:**
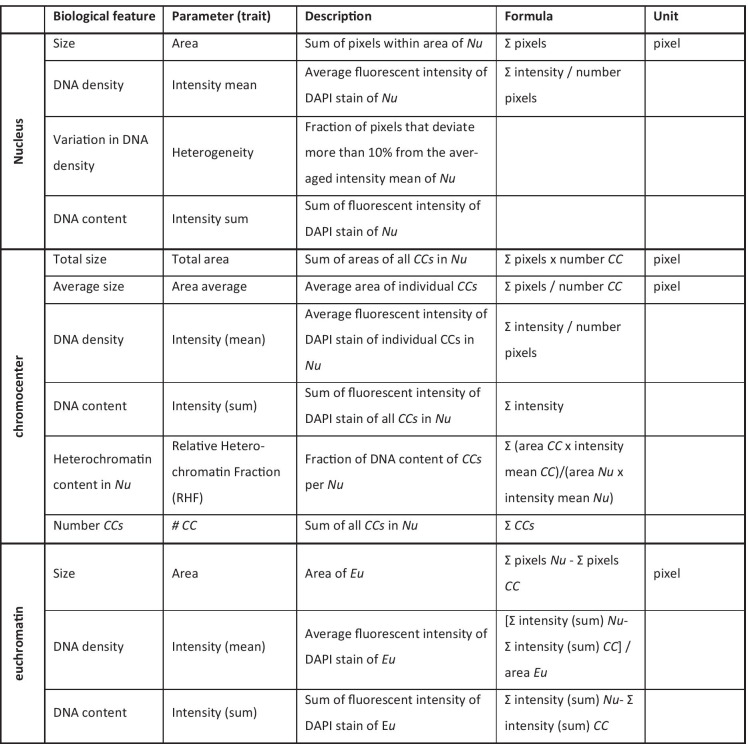
Morphometric parameters used for chromatin profiling together with their biological equivalent, description, and mathematical formula.

Note: Chromocenter (CC) is used in the meaning of a microscopically visual heterochromatin body in the interphase nucleus. DNA density as a biological feature is used as equivalent to DNA concentration.

*Nu* nucleus, *Eu* euchromatin.

**Table 2:**
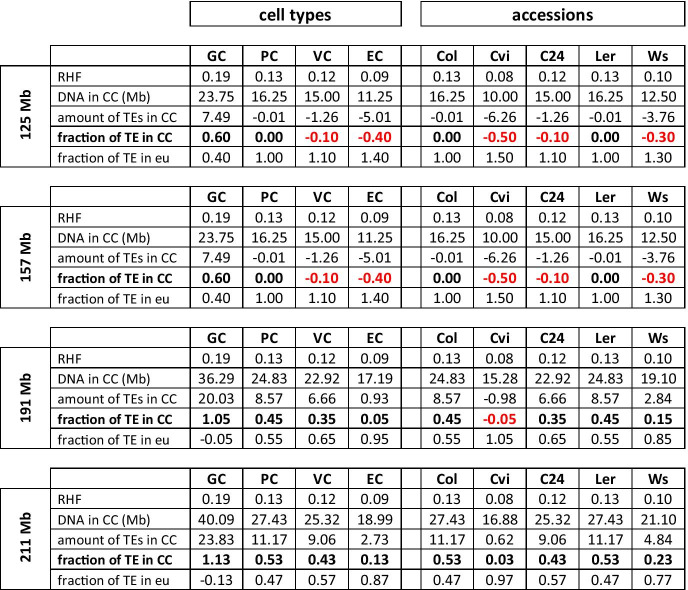
Fraction of transposable elements in heterochromatin and euchromatin.

*CC* = chromocenter, *TE* = transposon element, *eu* = euchromatin, *GC* = guard cell, *PC* = pave-ment/parenchyma cell, *VC* = vascular cell. *EC* = endopolyploid cells, *Mb* = megabasepairs.

Note: The negative values for the *fraction of TEs in CC* (red) suggests that in addition to all TEs also a fraction of the major tandem repeats is in euchromatin. The negative values for the *fraction of TEs in euchromatin *(i.e., GC values in 191 Mb and 211 Mb) suggests that more DNA in the CCs than the sum of tandem repeats and TEs. This implies that genes are also present in CCs.

For the calculation of the fraction of transposon elements (TEs) in chromocenters or euchromatin, we reasoned as follows. The total amount of TEs was estimated at 10% (Arabidopsis Genome Initiative 2000). The total amount of the centromeric 180 bp arrays was estimated at 8.76 Mb, based on pachytene FISH analysis (Haupt et al. 2001). For the total size of NOR domains, we used the value of 7.5 Mb based on CHEF gel analysis of the 45S ribosomal gene arrays (Copenhaver and Pikaard 1996). Although previous studies have shown significant copy number variation for the 45S ribosomal gene arrays, in particular from Swedish accessions (Long et al., 2013), the 45S rDNA copy number variation in the accessions used in our study corresponds to less than 1% of the total genome size (Woo and Richards, 2008). We did not include the 5S ribosomal repeats, spanning in total about 0.5 Mb (Campell et al., 1992), since this array is often found outside chromocenters (Mathieu et al., 2003; Tessadori et al., 2007a). The fraction of TEs in CCs is calculated as follows:

$$\mathrm{TE fraction in CC}=\frac{RHF\times \mathrm{Genome size}-\mathrm{total amount of }45\mathrm{S and }180\mathrm{ bp DNA}}{\mathrm{total amount of TE}}$$

The total amount of 45S and 180 bp DNA represents the amount of major tandem repeats (19.3 Mb). The product of *RHF* and genome size equals the amount of DNA in the CC. The total amount of TE is 10% of the genome size. The calculation also involves the genome size of Arabidopsis, which differs between several studies. Therefore, we used in this study four estimates for the genome size: 125 Mb (Arabidopsis Genome Initiative, 2000), 157 Mb (Bennet et al., 2003), 191 Mb (Doležel et al., 1998), and 211 Mb (Schmuths et al., 2004). The fraction of TEs in euchromatin is determined by subtracting the fraction TEs in CC from 1.

### Results and discussion

#### Comparison of software tools and DNA dyes for morphometric analysis

Accurate analyses of nuclear morphometric parameters rely on the usage of appropriate techniques and choices made, such as the preferred DNA stain, the image acquisition platform, and the computational image tools. In addition, quantification of heterochromatin requires proper segmentation of condensed, stained areas in the nucleus, whereby thresholding (and thus resolution and resolving power) is critical.

**Fig. 1:**
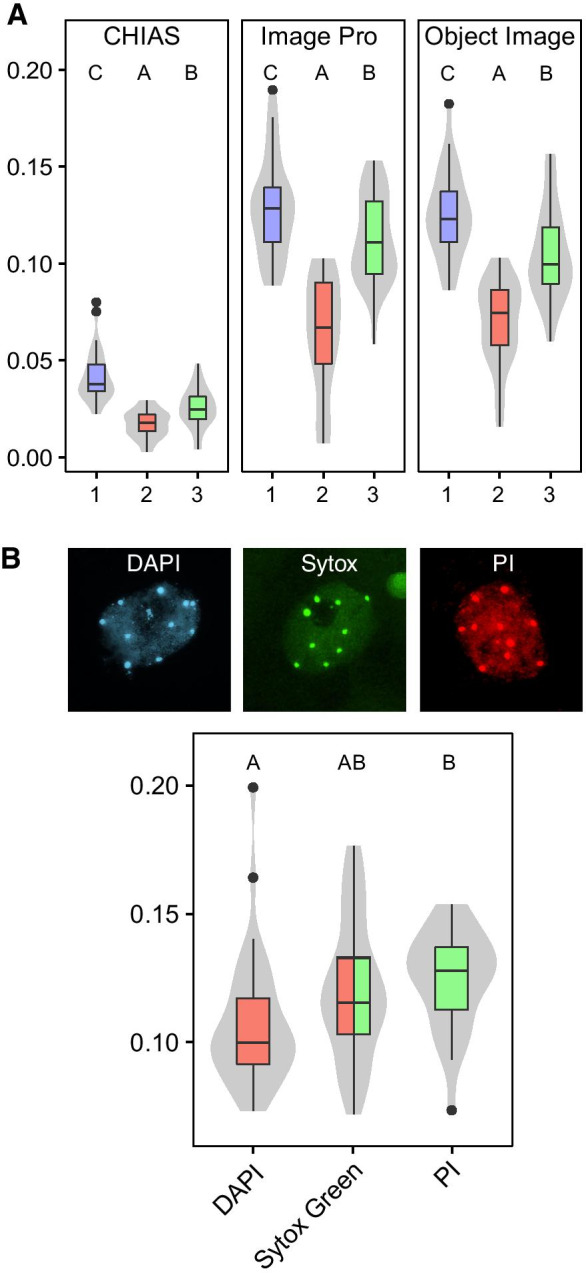
Morphometric analysis of isolated nuclei. **A** Whisker-box plots of *RHF* (*Y*-axis) from three independent sets of DAPI-stained L*er* nuclei (set 1, blue, *n* = 32; set 2, red, *n* = 31; set 3, green, *n* = 34), each of them analyzed with CHIAS, Image Pro + and Object Image. **B** Representative examples of nuclei (upper row) and whisker-box plots of *RHF* (Y-axis) from parenchyma/pavement nuclei (lower row), stained with 4′,6-diamidino-2-phenylindole (DAPI; blue), Sytox-Green (green), and propidium iodide (PI; red). Boxes indicate boundaries of second and third quartiles of data distributions. Black bars within the boxes indicate the median, and the error bars (whiskers) indicate the Q1 and Q4 values within 1.5 times the interquartile range. Observations outside 1.5 times the interquartile range are indicated as dots. Violin plots designate phenotype distributions. Letters (**A**, **B**, **C**) and colors indicate statistical differences between groups, with different letters indicating significantly different groups (*p* < 0.05) per panel.

We assessed three software packages; Object Image (Vischer et al. 1999), CHIAS (Fukui, 1986, 2005), and our in-house developed Image Pro + macro (Pavlova et al., 2010), for simple and rapid quantification of the relative heterochromatin fraction (*RHF*) of 2D images of interphase nuclei, to estimate their accuracy in data measurements. The *RHF* values of three independent sets of nuclei (~ 30 nuclei/set) with different heterochromatin compactions were determined with each software package (Fig. 1A). The values of the three sets displayed a similar pattern when analyzed by the three programs: set 2 was significantly lower than set 3 and that was significantly lower than set 1. Although the values for the sets 1, 2, and 3 obtained with Object Image and Image Pro + were comparable (*p* = 0.417), those with CHIAS were about four times lower (*p* < 0.01). These differences in *RHF* values can be explained by the way thresholding of the “objects” is determined. But overall, it is clear that the three image analysis programs produce comparable accurate measurements of relative differences in heterochromatin content between different datasets, if analyzed with the same software. Care should however be taken when comparing datasets obtained with different analysis (software) methods. In our experiments, we preferred to use the Image Pro + macro tool as it enables us to analyze large data sets with high accuracy and with a wide selection of quantification parameters.

 Nuclear morphometric analyses of 2D nuclei are based on pixel intensity measurements of stained (fluorescent) DNA. Diverse processes may affect the interaction between the dye and the DNA structure (e.g., *minor* or *major groove*) or the affinity of the dye for certain DNA (RNA) sequences (e.g., *sequences enriched AT content*) (Schweizer 1976). We therefore compared the *RHF* values obtained from nuclei stained with different DNA-specific fluorophores dyes: DAPI (emission: 461 nm, spatial resolution 0.24 µm), SYTOX®-Green (533 nm, 0.25 µm), and propidium iodide (636 nm, 0.33 µm), as these fluorophores are commonly used in DNA visualization, chromosome staining or flow cytometry (Williamson and Fennell, 1979; Russell et al. 1975; Fried et al. 1976; Hulett et al. 1969). Since PI has affinity to both DNA and RNA, we treated the slides with *RNase* prior to staining. Images of parenchyma and pavement nuclei were captured by fluorescent microscopy and the *RHF* was calculated with Image Pro + . We observed that mean values for *RHF* of Sytox green are 8% higher than that of DAPI, PI 14% higher than that of DAPI, and PI 5% higher than that of Sytox green (Fig. 1B). The differences were however not significant (ANOVA, *p* = 0.1). These slight differences might be explained by a combination of DNA/RNA, and AT/GC specificity, binding to both DNA and core histones (Banerjee et al. 2014) and wavelength dependent optical resolution of the objective. We decided to work with DAPI in the remainder of our experiments, because of slightly higher optical resolution, double-strand DNA specificity (no RNase treatment needed), relatively low fluorescence fading, and the limited cross-talk with other narrow-band fluorescence filter sets.

### Cell type–specific profiling of nuclear morphology

**Fig. 2:**
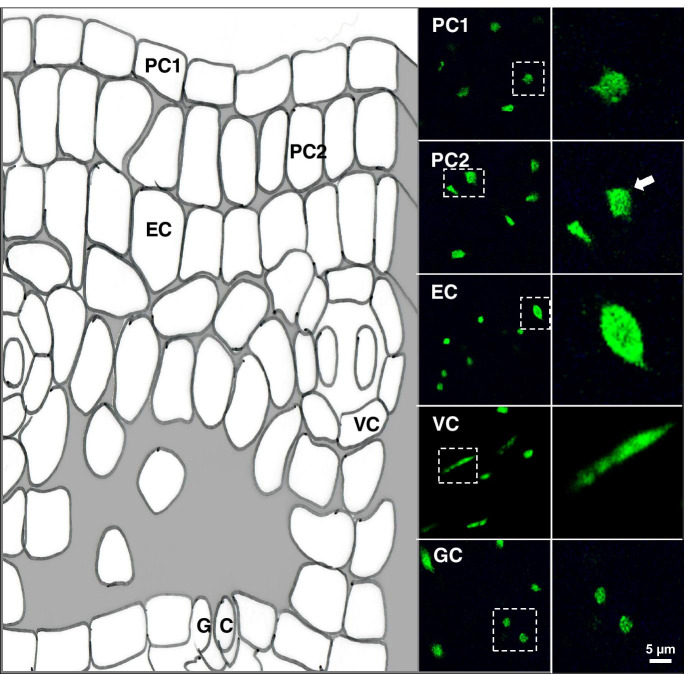
Identification of nuclei from different cell types of a young Arabidopsis leaf. On the left is a schematic drawing of a cross section obtained from a cross-sectioned leaf drawing (https://commons.wikimedia.org/wiki/File:Cross_section_of_Arabidopsis_thaliana,_a_C3_plant..jpg). On the right are images of H2B-YFP-stained nuclei derived from optical confocal sections of different cell types and selected magnifications. The right column represents a magnification of the boxed areas (white dashed lines) displayed in the left column. From top to bottom: pavement cell nucleus (PC1), parenchyma nucleus (PC2), endopolyploid cell nucleus, vascular cell nucleus (VC), and guard cell nucleus (GC). Depth of the *z*-stack is 16 μm. Bar for scale represents 5 μm.

Plant leaves change in composition and function during development. Since nuclei in differentiated, old leaves contain a high heterochromatin fraction (Tessadori et al. 2004) and exhibit increased endopolyploid levels (Del Prete et al. 2019), we decided to focus on young, ~ 5-mm-long rosette leaves of stage 1.05 (Boyes et al. 2001). Confocal microscopy images of transgenic plants expressing H2B-YFP protein (in the C24 genetic background) revealed clear differences in size and shape of nuclei between distinct cell types derived from different leaf tissues (Fig. 2). Nuclear isolation followed by spread preparation and DAPI staining yielded a mixed population of distinctive leaf nuclei. We sorted the nuclei based on nuclear size and nuclear shape and grouped them using microscopic observations as follows: (1) small and spherical, (2) medium-sized and elliptic/irregular shape, (3) elongated, (4) large. We assigned those as nuclei of (1) guard cell (GC), (2) parenchyma/pavement cell (PC), (3) vascular cell (VC), and (4) endopolyploid cell (EC). We based this classification on 3D data (Fig. 2) and information from the literature (Traas et al., 1998, Schubert et al., 2012, Del Prete et al., 2014, Poulet et al., 2017). However, based on this qualitative data alone, we cannot fully exclude that some parenchyma cells for instance have small, round nuclei. Of note, EC have replicated their DNA at least once, without undergoing cell division (Leitch and Dodsworth, 2017), although a diploid nucleus in G2-phase is difficult to distinguish from a tetraploid nucleus in G1.

**Fig. 3:**
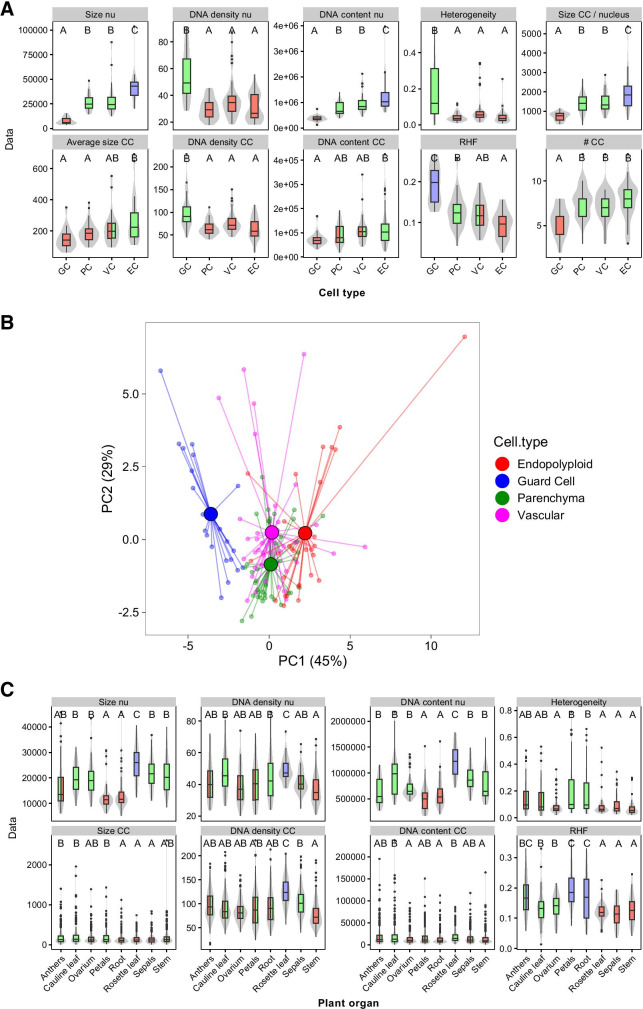
Morphometric profiling of nuclei from different cell types. Whisker-box plots showing morphometric differences between cell types **A** and between organs **C**. **B** Principal component analysis on scaled parameters in different cell types reveals four clusters of accessions, with the GC cluster more separate from the other three. Boxes indicate the boundaries of the second and third quartiles of the data distribution. Black bars within the boxes indicate the median, and the error bars (whiskers) indicate Q1 and Q4 values within 1.5 times the interquartile range. Observations outside 1.5 times the interquartile range are indicated as dots. Violin plots designate phenotype distributions. Significance levels are indicated as letters above the bars and represent a two-side *t*-test assuming unequal variances, with different letters indicating significantly different groups (*p* < 0.05) per panel. Colors of the boxes are shared if statistically similar. Units on the *Y*-axis are arbitrary.

Quantification of morphometric parameters (Table 1) revealed clear differences in nuclear features between the cell types (Fig. 3A). GC nuclei are the most remarkable with a round nuclear phenotype with a diameter of 4.84 µm. Their volume is estimated at 18 µm^3^, which matches the volume of the GC nucleus in 3D microscopic images (Poulet et al. 2017). Also, the size ratio between GC nuclei and PC nuclei is in agreement with other studies (Kato and Lam 2003; Poulet et al. 2017). Compared to the other cell types, GC nuclei contain about two times less *DNA content* and are 2–3 times smaller. They display the highest *DNA density* in euchromatin and in heterochromatin, which corresponds to a higher concentration of DNA. Guard cell nuclei also show the highest *RHF* (= 0.19). In comparison, the other cell types have *RHF* values varying from 0.095 to 0.13. The large CC/nucleus size ratio of GC cells further contributes to the typical morphological appearance of condensed chromatin. In addition, the high *heterogeneity* points at large variation in DNA compaction and endorses the sharp boundaries between the heterochromatin chromocenters and the surrounding euchromatin in the guard cell nuclei. In contrast, the PC, EC, and VC nuclei show less variation in *heterogeneity*, suggesting a more uniform distribution of DNA inside the nucleus. Additionally, the relatively low number of distinguishable chromocenters (5.5 per nucleus) implies that chromocenters are frequently associated in guard cell nuclei. The high DNA density in GC nuclei approximates the DNA density in chromocenters of PC nuclei and points to high chromatin compaction in the GC nucleus. This may have consequences for gene activity, since compaction leads to reduced mobility and hence to less interaction between distal chromosomal regions. Indeed, Kato and Lam (2003) detected with LacI-GFP that the confinement areas of the tagged loci with repetitive LacO arrays are six times smaller in GC nuclei compared to PC nuclei. Hence, the chromatin movement area in GC nuclei is apparently restricted, which is in accordance with the high chromatin compaction in the GC nucleus in our study.

The product of *DNA density* and *area* represents an estimate of *DNA content* from which the approximate C-value of the nuclei can be calculated. We worked from the assumption that guard cell nuclei have a DNA level of 2C (Melaragno et al. 1993) and estimated that pavement and parenchyma cells have on average 4C, vascular cells 5C, and endopolyploid cells 6.7C. Consequently, the majority of the diploid GCs are in the G_0,1_ phase of the cell cycle and are not endoreduplicated, while PC nuclei are predominantly in G_2_, although endoreduplication cannot be excluded. The polyploid DNA levels in PC, VC, and EC and their lower DNA density (compared to GC) suggest more chromatin accessibility in cells with higher ploidy levels. A similar correlation between chromatin conformation and ploidy level was reported in other studies (Kato and Lam, 2003; Schubert et al., 2012). The higher accessibility of chromatin in polyploid nuclei suggests that more gene copies in endoreduplicated cells facilitate more transcripts in specialized cells.

**Fig. 4:**
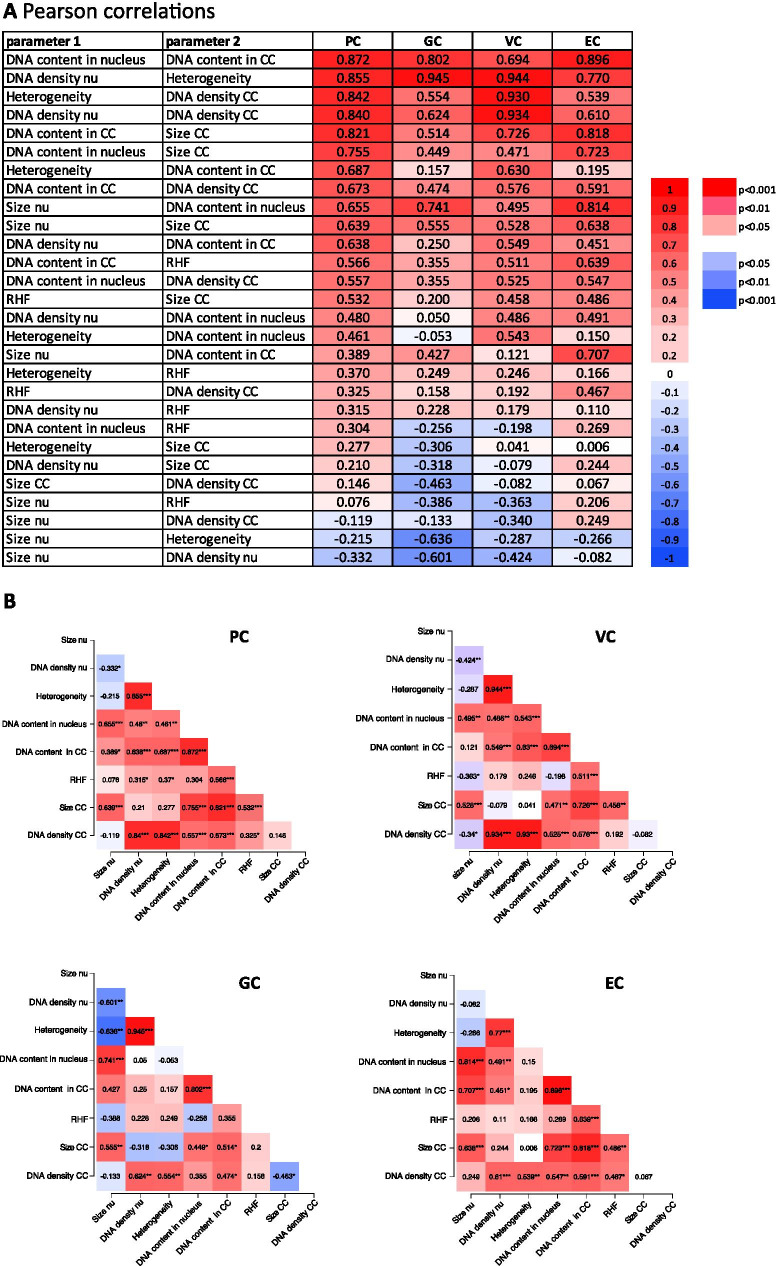
Pearson correlation between parameters in different cell types. **A** Heatmap and **B** correlation matrix showing the pairwise correlation coefficients **A** and significance of correlations **B** between and among nuclear and chromocenter parameters. Color intensity indicates the strength of the correlation **A** and significance levels **B**. Positive correlations are indicated in red, negative correlations, in blue (see legend). Ranking is according to PC values. Cc, chromocenter; TE, transposon element; GC, guard cell; PC, pavement cell; VC, vascular cell; EC, endopolyploid cells. *p < 0.05; **p < 0.01; ***p < 0.001.

We subsequently examined correlations between the morphometric parameters in a pairwise manner and generated correlation heatmaps (Fig. 4A) and matrices (Fig. 4B) for each cell type. Since euchromatin is the prevailing chromatin fraction in the *Arabidopsis* nucleus (> 80%), we did not include euchromatin parameters and focused on the correlation between nuclear (nu) and chromocenter (CC) parameters. The amount of DNA in the nucleus is significantly (*p* < 0.001) related to the *DNA content* in CCs, which suggests that the quantitative partition of DNA into euchromatin and chromocenters is maintained at higher ploidy levels. The pairwise correlation shows that nuclei of guard cells are different compared to nuclei from the other cell types in having weaker correlations between the morphometric parameters. In some cases, GC nuclei show greater similarity to EC. For example, *heterogeneity* is significantly related to *DNA density* in chromocenters of PC and VC (coeff. 0.8–0.9, *p* < 0.001), but less, yet still significantly, in GC and EC (coeff. 0.5–0.6, *p* < 0.01). Heterogeneity is a measure for the variation in DNA density in euchromatin and heterochromatin and, hence, is more dependent on the level of DNA condensation in both chromatin domains. For *DNA density* in the nucleus and *DNA density* in chromocenters, we observed the expected similar high levels of correlation in PC and VC vs less in GC and EC. This may be explained by the fact that PC and VC show similar values for most of the nuclear parameters, whereas GC and EC are often significantly different (Fig. 3A). A principal component analysis of the four groups of nuclei revealed four different clusters in which the GC cluster is more separated from the other three clusters and more opposite to the EC cluster (Fig. 2B).

In order to assess intraspecific morphometric and genetic variation in nuclear and chromatin phenotypes, we quantified morphometric parameters of nuclei isolated from different organs, i.e., anthers, cauline leaf, ovarium, petals, root, rosette leaf, sepals, and stem. As expected, many parameters varied between the different organs, such as *size*, *DNA density*, *DNA content,* and *heterochromatin content* (*RHF*) of the nucleus and *DNA density* of the CC (Fig. 3B). Nuclei of anthers, petals, and roots have the smallest nuclei, the highest *RHF*, and the lowest *DNA content*, with almost similar values as guard cells, although these tissues lack guard cells (Pillitteri et al. 2008). The nuclear phenotypes of the organs reflect the presence of mixed tissues with different cell types. In conclusion, our morphometric analysis shows that measuring *area* and pixel intensities of flattened 2D nuclei preparations and components thereof (*i.e.*, chromocenters) allows profiling of different types of nuclei and enables detailed analysis of dynamic interrelated nuclear features.

### *Arabidopsis* accessions differ in nuclear morphometry

**Fig. 5:**
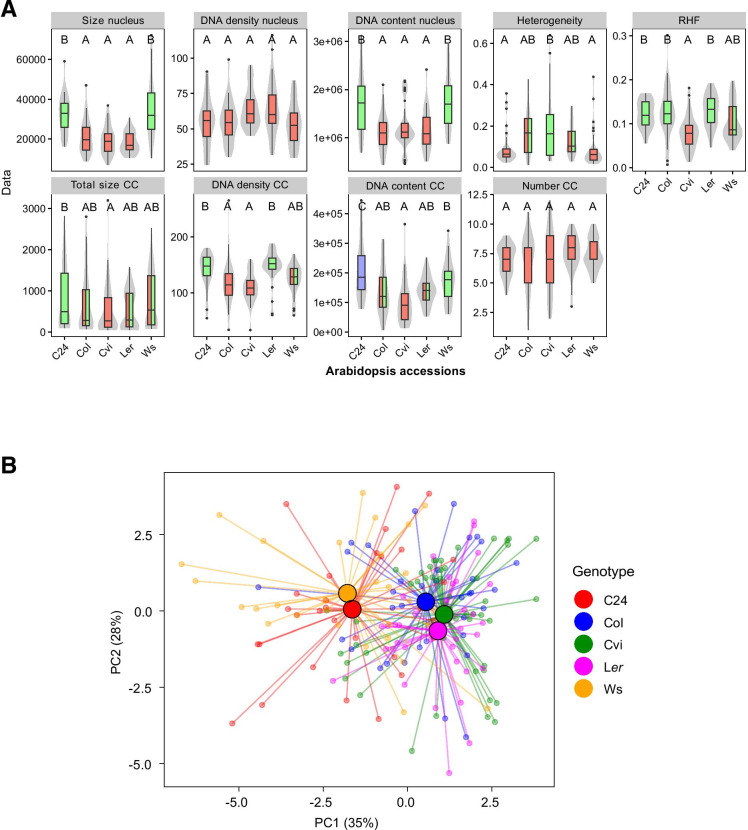
Morphometric profiling of PC nuclei from different accessions. **A** Whisker-box plots showing morphometric differences between accessions. Boxes indicate the boundaries of the second and third quartiles of the data distribution. Black bars within the boxes indicate the median, and the error bars (whiskers) showing the values in Q1 and Q4 within 1.5 times the interquartile range. Observations outside 1.5 times the interquartile range are indicated as dots. Violin plots designate phenotype distributions. Significance levels are indicated as letters above the bars and represent a two-side *t*-test assuming unequal variances. Different letters indicate significant differences (*p* < 0.01) per panel. Colors of the boxes are shared if statistically similar. Units on the *Y*-axis are arbitrary. **B** Principal component analysis on scaled parameters in different accessions reveals two clusters of accessions.

*Arabidopsis thaliana* is native to diverse climates throughout the Northern hemisphere and accessions have adapted to their local environment (Hancock et al., 2011). We previously demonstrated that chromatin compaction values correlate with geographical latitude of origin within a diverse panel of natural accessions (Tessadori et al. 2009). Subsequent genetic analyses indicated that light intensity is a major determinant of chromatin (de)condensation (Tessadori et al. 2009; van Zanten et. al. 2010; Snoek et al. 2017). Here we extended on this analysis, by considering nuclear morphometry using nuclei from parenchyma and pavement cells (PC) of the accessions C24, Col, Cvi, L*er*, and Ws-2. A significant difference was found in the *RHF* (*p* < 0.01) between the accessions, with Col and L*er* having the highest *RHF* (0.13) while the lowest *RHF* (0.08) is found for *Cvi* (Fig. 5A). These observed *RHF* values match well with the results from our previous studies on *RHF* (Soppe et al. 2002; Tessadori et al. 2007a, b; Snoek et al. 2017) and HX (Tessadori et al., 2009).

Overall, the accessions C24 and Ws show comparable morphometric features, including *nuclear size*, *nuclear DNA content, DNA content in CCs*, and *heterogeneity*, in which they differ from the other accessions. The two accessions differ however from each other in *DNA density in CCs* and for the compound trait *RHF*, which is significantly lower in Ws-2 than in C24 (Fig. 5A). This indicates that small differences in individual parameters can have a profound effect on overall nuclear appearance, when combined. The relatively high *DNA content* suggests that C24 and Ws have more PC nuclei in the G2 or early endoreduplicated phase of the cell cycle (= 4C) than the Col, Cvi, and L*er* accessions under the tested conditions and developmental state. The ~ 1.5 times larger nuclear volume of C24 and Ws supports this conclusion. A principal component analysis of the five accessions assigned C24 and Ws to a cluster and Col, Cvi, and L*er* to another cluster, further supporting the similarity between C24 and Ws based on the morphometric features (Fig. 5B).

**Fig. 6:**
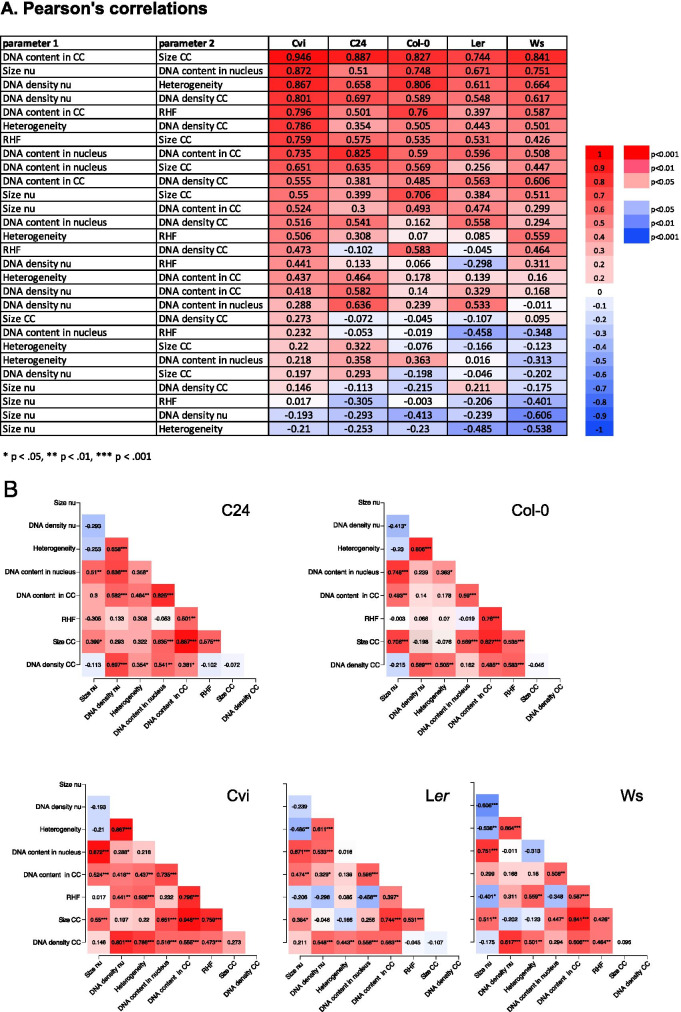
Pearson correlation between parameters in different accessions. **A** Heatmap and **B** correlation matrix, showing the pairwise correlation coefficient between and among nuclear and chromocenter parameters of the five tested accessions. Color intensity indicates the strength of the correlation **A** and significance levels **B**. Positive correlations are indicated in red, negative correlations, in blue (see legend). Ranking is according to PC values. Cc, chromocenter; TE, transposon element; GC, guard cell; PC, pavement cell; VC, vascular cell; EC, endopolyploid cells. *p < 0.05; **p < 0.01; ***p < 0.001

Subsequently, we generated correlation heatmaps and matrices (Fig. 6) to visualize correlations between morphometric parameters for each accession. The number of chromocenters, which is similar (7–8) for all accessions, is not or only weakly correlated to the other parameters (Suppl. Figure S2). Apparently, the process of chromocenter association in parenchyma/pavement cells occurs independently of DNA content or size of the nucleus. Overall, the correlations between *nuclear size* and *DNA content* are strong in Cvi, moderate in Ws, Col and L*er*, and weak in C24. Since the feature *DNA density*, which is equivalent to DNA concentration, can be defined as DNA amount per volume, the correlation between *nuclear size* and *DNA content* can be reflected in the *DNA density* parameter. An increase in *DNA content*, for example, by DNA replication, enlarges the *nuclear size*, but not the *DNA density* in Cvi, Ws, Col, and L*er*. In contrast, in C24, an increase in *DNA content* is accompanied with a moderate increase of the *DNA density* and a weak increase of the *nuclear size*. The C24 accession also differs from the other accessions in having a sharp boundary of the chromocenters. In comparison, Ws-2 and Cvi exhibit more diffuse CCs showing a more gradual transition between heterochromatin and euchromatin.

All accessions show strong correlations (coeff. > 0.84) between *CC size* and *DNA content of CC*, which suggests that an increase in heterochromatin does not primarily results in more compaction of CCs, but rather in enlargement of the CC domains. Hence, the observed values for the *DNA density* in CC is possibly the upper limit in PC cells of these accessions. *DNA density* in CCs was strongly correlated (coeff. = 0.80) only with *nuclear DNA density* in Cvi. This accession has the lowest *DNA density* in CC (Fig. 5A) and also displays the lowest *RHF* value, probably due to “low light stress” that this sub-tropical accession (latitude 15.1 ^o^N) experiences under our greenhouse conditions of the relatively northern city of Amsterdam (latitude 52.4 ^o^N) (Tessadori et al. 2009). Interestingly, Cvi is the accession with overall the strongest correlations, showing significantly high levels of pairwise correlations between many nuclear parameters (Fig. 6A). In contrast, relatively few parameter correlations are found in L*er*. This may indicate that the nuclear phenotype of L*er* is more robust: a change in a certain parameter has limited effect on another parameter. The high correlation values in Cvi indicate that changes in several parameters may influence each other and suggest that nuclear organization in Cvi is more plastic in the tested conditions.

**Fig. 7:**
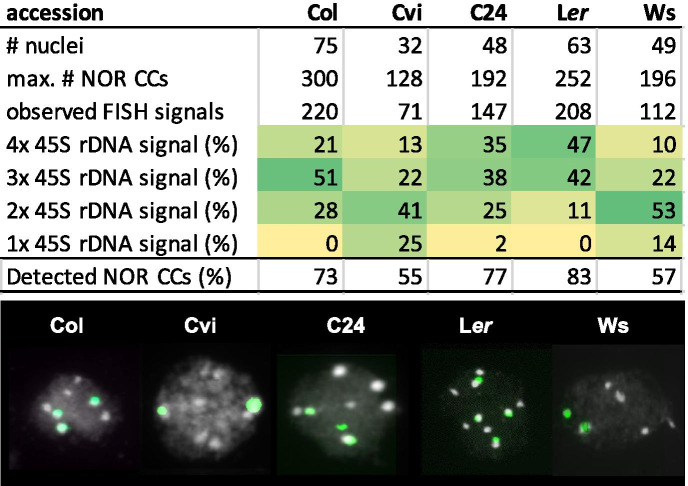
Quantification of NOR-chromocenter association in Col, Cvi, C24, L*er*, and Ws. The low percentages of detected NOR-CCs in Cvi and Ws indicates a high association of NOR-CCs. Lower panel shows representative examples of the 45S rDNA FISH signals in PC nuclei of the four accessions.

Chromocenters CC2 and CC4 accommodate nuclear organizing regions (NORs) containing ribosomal gene arrays and associate most frequently with each other or with other CCs (Fransz et al. 2002; Pecinka et al. 2004). To elaborate on this result and the observed striking variation in CC condensation between the accessions, we tested for a correlation between nuclear morphology and the association of CC2 and CC4. We applied FISH with a 45S rDNA probe and counted the number of fluorescent signals per nucleus (Fig. 7). The results indicate that accessions have different preferences for association of the NOR CCs. Ws and Cvi displayed significantly less 45S rDNA signals than the other tested accessions. These two accessions also exhibit lower *RHF* values compared to Col, L*er*, and C24. Considering a similar average number (7–8) of chromocenters in all accessions, it is therefore likely that in Ws-2 and Cvi, the NOR chromocenters are preferentially associated. Interestingly, these two accessions have a much lower level of rDNA methylation compared to Col, L*er*, and C24 (Woo and Richards, 2008), which is in line with their low *RHF*. These data suggest a mechanistic link between DNA methylation level and the association of heterochromatic NOR domains. We speculate that less DNA methylation of tandem repeats correlates with more association of the corresponding heterochromatin domains.

### Fraction of TEs in CC and euchromatin

The *RHF*, which represents the fraction of genomic DNA in CCs, allows to estimate the fraction of transposable elements (TEs) in chromocenters. From previous studies, it is known that long tandem repeat arrays, such as ribosomal gene regions and the centromeric 180 bp repeat regions, are stable components of CCs, while transposon-rich regions and 5S rDNA repeats can easily disperse from the CC territories (Mathieu et al., 2003; Tessadori et al. 2007a, b). The long tandem repeats 45S rDNA and 180 bp decondense only under exceptional conditions, such as complete dedifferentiation during protoplast isolation (Tessadori et al., 2007a), or in the strong chromatin remodelling mutant *ddm1-5* (Mittelsten Scheid et al. 2002). It follows that the *fraction of TEs in CCs* can be estimated by subtracting the DNA representing the long tandem repeats from the total DNA present in the CC (see M&M for the calculation). We calculated the *fraction of TE in CC* for the group of cell types and the group of accessions (Table 2). The *fraction of TE in the CC* is highest in GC nuclei, which is not surprising, since these cells have the highest *RHF* (0.19). Remarkably, in several cell types and accessions, we observe a negative value for the *fraction of TE in CC*, which is the case when the amount of DNA in CC (= genome size x *RHF*) is lower than the amount of major tandem repeats (= 16.3 Mb). A combination of a low *RHF* and a low genome size is responsible for the negative values of the *fraction of TE in CC*. This negative value implies that not only all TEs are outside the CCs, but also a fraction (several megabases) of the major tandem repeats is no longer part of heterochromatin. We have never observed decondensation of major tandem repeats in plants under normal conditions, but only under high stress, such as protoplast formation or in the mutant *ddm1-5* (Tessadori et al., 2007a; Mittelsten Scheid et al., 2002). Therefore, we suggest that the genome size is too low if the *fraction of TE in CC* becomes less than zero in plants grown under non-stress conditions. This is the case for the genome sizes 125 Mb, 157 Mb, and perhaps 191 Mb. Only for the estimated genome size 211 Mb did we find no negative values for the *fraction of TE in CC*.

In case the genome size of Arabidopsis is 211 Mb, we can infer that in PC (in Col, L*er*, and C24), VC, and GC a significant fraction (> 40%) of TEs is found in the CCs. The only cell type that did not show a negative value for the fraction of TE in CC for any of the genome sizes is the GC, which has the highest *RHF* (0.19). For the GC, we calculated a TE fraction (1.13) in CCs. This fraction is greater than 1.0, which implies a DNA amount in CCs that is higher than the total amount of genomic repeats (i.e., tandem repeats plus TEs). We conclude from this that in guard cells, not only the tandem repeats and all TEs is allocated in the CCs, but also a substantial fraction of gene-rich regions. These results match with the observation that guard nuclei have highly condensed chromatin, both in euchromatin and in CCs (see above).

The endopolyploid cells appear to have the majority (87%) of TEs in euchromatin, which supports the idea that endoreduplicated nuclei have more accessible chromatin for transcription. The PC nuclei of the accession Cvi has the highest percentage (97%) of TEs in euchromatin domains, which is in agreement with the highly decondensed appearance of this accession under our climate chamber conditions (Tessadori et al., 2009). Ws also displays a major fraction (77%) of TEs outside the chromocenters. Assuming a genome size of 211 Mb, we estimate that all repeats (tandem arrays and TEs) are in CCs when the *RHF* is 0.18 or higher while all TEs are in euchromatin when the *RHF* is 0.07 or lower (Suppl Table S1).

## Conclusions

Our microscopic study demonstrates that 2D image analysis based on pixel number and DAPI fluorescence intensity enables detailed assessment of nuclear morphometric profiles of different cell types and accessions. These profiles shed light on genomic elements in the nuclear context, elucidating the obvious compaction of gene regions into chromocenters of guard cell nuclei and the decondensation of a greater part of the transposons in endopolyploid cells of Col and in parenchyma and pavement cells of Cvi. Differences between cell types and accessions may be related to genomic and epigenetic variation in the rDNA loci between the accessions. The type of morphometric analyses and correlation profiling as presented in this study constitute an important step towards further local and global analysis on gene activity, DNA methylation in ribosomal genes, and assessment of robustness of biotic and abiotic stress tolerance in genomes. Therefore, we propose morphometric analysis as a swift and easy tool to estimate genome integrity of plant subjected to environmental stress.

## Supplementary Information

Below is the link to the electronic supplementary material.

Supplementary file1 (DOCX 22 KB)

## Data Availability

Not applicable.

## Abbreviations

CC: Chromocenter
DAPI: 4’,6-Diamidino-2-phenylindole
EC: Endopolyploid cell
Euchr: Euchromatin
GC: Guard cell
HX: Heterochromatin index
NOR: Nucleolar organizer region
PC: Pavement/parenchyma cell
PI: Propidium iodide
*RHF*: Relative heterochromatin fraction
TE: Transposable element
VC: Vascular cell
