# Supplementary material for: 2D morphometric analysis of Arabidopsis thaliana nuclei reveals characteristic profiles of different cell types and accessions
Source: Chromosome Res. 2021 Oct 19;30(1):5–24. doi: 10.1007/s10577-021-09673-2 (PMC8942920; doi:10.1007/s10577-021-09673-2)
Supplement: Supplementary file 1 — Supplementary file1 (DOCX 22 KB) [file 10577_2021_9673_MOESM1_ESM.docx]

**Supplementary material**

RHF macro for Image Pro Plus, v. 5 (*Pavlova et al. 2010*)

Option Explicit

Sub nuclei quantification() *Determine which parameters are static (string) and which are dynamic (integer)*

Dim filename3 As String*100

Dim imname As String

Dim imdir As String

Dim lenname As Integer

Dim outlinename As String

Dim i As Integer

Dim infname As String*255

Dim iname2 As String

Dim more As Integer

Dim imdir2 As String

Dim file As Integer

Beginning:

ret= ipappcloseall()

file = IpStGetName("Find the file you want to process","c:\my documents\nuclei images", "*.tif", Filename3) *A folder with images was chosen*

If file = 0 ThenMsgBox ("please select a file to analyze")

GoTo Beginning

End If

imdir = CurDir(filename3)

imdir2 = imdir

i=0

retry:

more = ipstsearchdir(imdir2, "*.tif",i, infname)

Debug.Print more

If more = 2 Then

i=i+1

GoTo retry

End If

ret = IpAppRun("C:\Program Files\Microsoft Office\excel.exe", RUN_MINIMIZED, RUN_AUTOCLOSE) MS

*Excel is supposed present and will be opened automatically for output data*

ret = IpDde (DDE_OPEN, "excel", "sheet1")

ret = IpDde(DDE_SET, "row", "1")

ret = IpDde(DDE_SET, "col", "1")

ret = IpBlbSaveData("c:\Documents and Settings\nuclei images\",S_DATA+ S_APPEND+S_Y_AXIS

+S_DDE)

Do While more = 1

imname = Dir (infname)

imdir = "c:\Documents and Settings\nuclei images\"

lenname =Len(imname)

Debug.Print lenname

outlinename = Left(imname,lenname-4)

ret = IpWsLoad(INFNAME,"tif") Image in TIFF format is converted into 8 bit grey scale (Figure 1a) and then duplicated

ret = IpWsConvertImage(IMC_GRAY, CONV_SCALE, 0, 0, 0, 0)

ret = IpWsDuplicate()

ret = IpHstEqualize(EQ_BESTFIT) *Mask the brightly fluorescing chromocenters with the Equalize command (Figure 1b, c)*

ret = IpLutApply()

*The* Best Fit option *was chosen to enhance the contrast and the dynamic range of the active image, by stretching the histogram between the minimal value black (0) and maximal value white (255)*

The Apply LUT (Lookup Table) command will reveal regions of enhanced contrast Blur the chromocenters with a median filter (7 x 7 kernels)
Use threshold and segmentation to find the Region Of Interest (is here the whole nucleus*)*

ret = IpFltMedian(7, 5)

ret = IpBlbShow(1)

ret = IpBlbSetAttr(BLOB_AUTORANGE, 0)

ret = IpBlbEnableMeas(BLBM_ALL, 0)

ret = IpBlbSetAttr(BLOB_CLEANBORDER,1)

*Measure of following parameters:*

ret = IpBlbEnableMeas(BLBM_AREA, 1) *Area (area of object)*

ret = IpBlbEnableMeas(BLBM_AREAPOLY, 1) *Area polygon (area included in the polygon defining the perimeter of the object’s outline)*

ret = IpBlbEnableMeas(BLBM_SIZECOUNT, 1)

ret = IpBlbEnableMeas(BLBM_DENSITY, 1) *Density mean (average intensity of the object)*

ret = IpBlbEnableMeas(BLBM_DENSSUM, 1) *Density sum (sum of intensity inside the object)*

ret = IpBlbEnableMeas(BLBM_HETEROGENEITY, 1)

ret = IpBlbEnableMeas(BLBM_MARGINATION, 1)

ret = IpBlbEnableMeas(BLBM_PERIMETER, 1) *Perimeter (length of the object’s outline)*

ret = IpBlbEnableMeas(BLBM_ROUNDNESS, 1)

ret = IpBlbEnableMeas(BLBM_SRANGE, 1)

ret = IpTemplateMode(1)

ret = IpSegShow(1)

ret = IpSegSetRange(0,100, 255)

ret = IpTemplateMode(0)

ret = IpBlbCount()

ret = IpBlbUpdate(0)

ret = IpTemplateMode(0)

ret = IpBlbHideObject(0,0,0)

ret = IpTemplateMode(0)

ret = IpBlbUpdate(4)

ret = IpBlbSaveOutline(imdir2 & "\" & outlinename & "nuclei.scl")

*Outline the ROI (Figure 1e) and output to MS Excel, and save the data*

ret = IpBlbSaveOutline(imdir2 & "\" & outlinename & "nuclei.out")

ret = IpSegCreateMask(2,0,1)

ret = IpSegShow(0)

ret = IpBlbLoadOutline(imdir2 & "\" & outlinename & "nuclei.scl")

ret = IpAppSelectDoc(1)

ret = IpOpImageLogic(3, OPL_AND, 1)

ret = IpBlbLoadOutline(imdir2 & "\" & outlinename & "nuclei.scl")

ret = IpBlbUpdate(4)

ret = IpBlbMeasure()

ret = IpDde (DDE_OPEN, "excel", "sheet1")

ret = IpBlbSaveData ("", S_APPEND+S_HEADER+S_Y_AXIS+S_DDE)

ret = IpAppSelectDoc(4)

ret = IpWsDuplicate()

ret = ipfltsobel() *Threshold and find the edges of the chromocenters (CCs)*

ret = IpFltShow(0)

ret = IpFltClose(MORPHO_2x2SQUARE, 10)

ret = IpFltErode(MORPHO_2x2SQUARE, 3)

ret = IpBlbShow(1)

ret = IpBlbSetAttr(BLOB_AUTORANGE, 1)

ret = IpBlbSetAttr(BLOB_BRIGHTOBJ, 1)

ret = IpAppSelectDoc(3)

ret = IpBlbCount()

ret = IpTemplateMode(0) *Perform a segmentation step and draw the outlines around the chromocenters*

ret = IpBlbHideObject(0,0,0)

ret = IpTemplateMode(0) *Number the outlined CCs from the top left corner to the bottom right corner and save the outline data of the CCs*

ret = IpBlbUpdate(4)

ret = ipappmenuselect(1, 5 , "", DLG_Menu_coord)

ret = IpBlbUpdate(4)

ret = IpBlbSaveOutline(imdir2 & "\" & outlinename & "chromocentre.scl")

ret = IpBlbSaveOutline(imdir2 & "\" & outlinename & "chromocentre.out")

ret = IpBlbMeasure() *Measure the same parameters as for the nucleus on the CCs*

ret = IpDde (DDE_OPEN, "excel", "sheet1") *Automatically export the data to Microsoft excel (or to the Clipboard)*

ret = IpBlbSaveData ("",S_APPEND+S_Y_AXIS+S_DDE)

ret = IpDde(DDE_SET, "row_inc", "1")

ret = IpDde(DDE_PUT, "r[-4]c", Chr$(34) & INFname & Chr$(34)) *Place the names of the analysed file 2 rows above the exported data*

ret = IpDde(DDE_SET, "append", "1")

i=i+1

more = ipstsearchdir(imdir2, "*.tif",i, infname)

ret= ipappcloseall()

Loop

ret = IpAppRestore()

End Sub
